# Evaluation of the Effect of Wheat Germ Oil and Olmutinib on the Thioacetamide-Induced Liver and Kidney Toxicity in Mice

**DOI:** 10.3390/life12060900

**Published:** 2022-06-15

**Authors:** Salman Alamery, Seema Zargar, Fatimah Yaseen, Tanveer A. Wani, Abdulaziz Siyal

**Affiliations:** 1 Department of Biochemistry, College of Science, King Saud University, Riyadh 11495, Saudi Arabia; salamery@ksu.edu.sa (S.A.); 438204071@student.ksu.edu.sa (F.Y.); 2 Department of Pharmaceutical Chemistry, College of Pharmacy, King Saud University, Riyadh 11451, Saudi Arabia; twani@ksu.edu.sa; 3 Department of Anatomy, College of Medicine, King Khalid University Hospital, Riyadh 11451, Saudi Arabia; drazizsiyal@gmail.com

**Keywords:** olmutinib, wheat germ oil, thioacetamide, liver toxicity, kidney toxicity

## Abstract

Thioacetamide (TAA) intoxication produces a reproducible standard animal model of induced liver and kidney injuries where free radicals are produced by phase I oxidation reactions, which eventually leads to liver and kidney failure. Wheat germ oil (WGO) is a unique food supplement with concentrated nutrient efficiency and has remarkable antioxidant functions. Olmutinib, on the other hand, is a chemotherapy drug considered safe for kidneys and the liver. Therefore, in this study, WGO and olmutinib were investigated for their effect on TAA-induced liver and kidney damage. Inflammatory markers; interleukin-1 beta (IL-1β); IL-6; and the levels of enzymatic markers ALT (Alanine aminotransferase), AST (Aspartate aminotransferase), LDH (Lactate dehydrogenase), and CK (creatinine kinase) in serum for liver and kidney were analyzed and evaluated along with histopathological changes in the tissue. Thirty male mice 4–6 weeks of age were grouped into five groups of six animals: the control group (saline) and the other groups (Groups II to V), which were given thioacetamide for two weeks. In addition, Group II continued with TAA; Group III was given olmutinib (30 mg/kg), Group IV was given the wheat germ oil (WGO) (1400 mg/kg), and Group V was given (olmutinib (30 mg/kg) + WGO (1400 mg/kg)) for five days. The results suggested that olmutinib treatment potentiated TAA-induced liver and kidney injury. At the same time, WGO efficiently alleviated TAA and TAA–olmutinib toxicity in Groups IV and V. The histological studies also showed reduced damage with WGO in the animal model. Hence, it was concluded that WGO could significantly reduce liver and kidney damage caused by TAA and olmutinib in mice.

## 1. Introduction

Liver and kidney diseases and injuries are the leading cause of mortality worldwide. The liver is responsible for more than five hundred vital body functions such as albumin production, bile production, blood filtration, glucose regulation, amino acid regulation, blood clotting, storage of amino acids, metabolism, gluconeogenesis, etc. [1,2,3]. In addition, the kidneys act as essential filters for waste and toxic substances, purifying our blood and reabsorbing vital minerals and vitamins into the bloodstream. Therefore, drug-induced injuries or damage to these vital organs responsible for maintaining homeostasis need to be explored for the protective mechanisms of natural supplements [4]. Furthermore, drug-induced organ injuries are distinctive and have different clinical or histological signatures [1]. The injuries or side effects may be due to the generation of reactive metabolites generated by metabolic phase I oxidation reactions. The generated reactive species have been shown to cause hepatotoxicity and renal damage [5,6,7].

Thioacetamide (TAA) (C_2_H_5_NS) is a well-known toxicant that causes centrilobular necrosis and fibrosis to internal organs such as the liver and kidney. TAA intoxication produces a reproducible standard animal model of fibrosis and cirrhosis in rodents [8,9,10,11,12]. In addition, TAA induces fibrosis and cirrhosis in organs other than the liver and kidneys, such as the lungs, intestine, thymus, spleen, and pancreas [13,14,15,16].

The TAA toxicity mechanism is attributed to thioacetamide sulfoxide (TAAS) and the subsequent transformation of TAAS to thioacetamide dioxide (TAAD). The TAAD biotransformation is less effective than TAAS biotransformation. Both the metabolites eventually lead to TAA-induced kidney and liver injuries within 6 to 12 h after TAA incorporation [17]. These TAA metabolites are reported to bind covalently to liver and kidney macromolecules and lead to increased reactive oxygen species generation [18]. This process follows with the induction of both lipid peroxidation and oxidative stress in both tissues. In addition, the covalent binding of TAA metabolites to antioxidant enzymes leads to decreased antioxidant potential in both organs [19]. Olmutinib is a third-generation chemotherapeutic drug and an EGFR tyrosine kinase inhibitor (TKI) used to treat non-small cell lung cancer. It competitively inhibits a transmembrane glycoprotein with an extracellular epidermal growth factor (EGFR) binding domain and an intracellular tyrosine kinase (TK) domain. Both domains control the processes of cell proliferation and apoptosis by regulating several downstream signaling pathways [17]. Olmutinib is considered safe for the kidney and the liver [18,19,20]. In addition, olmutinib is used to manage patients sensitive to other TKIs such as erlotinib and neratinib [21].

Furthermore, wheat germ oil (WGO) has a high content of potent natural antioxidants and is one of the high-quality food sources of vegetable proteins. It has ample pharmaceutical applications, protects against hepatotoxicity, and inhibits lipid peroxidation induced by various superoxide-free radicle producers [22,23]. In addition, wheat germ has various beneficial effects due to benzoquinones; 2-methoxy benzoquinone; and 2,6 dimethoxy benzoquinone [24]. Despite its unlimited health benefits, WGO is rarely used for human nutrition. Avemar is a non-toxic wheat germ extract and has been approved clinically in Hungary since 1998 and in the Czech Republic, Bulgaria, and Romania as a medical nutrient for cancer patients [25]. Our study investigated the impact of olmutinib and WGO on TAA-induced mice inflammatory and enzymatic markers such as interleukin-1 beta (IL-1β) and IL-6 changes in the liver and kidney tissues of mice. In addition, the kidney and liver markers from serum *viz* ALT (Alanine aminotransferase), AST (Aspartate aminotransferase), LDH (Lactate dehydrogenase), and CK (creatinine kinase) were evaluated along with tissue histopathological examination.

## 2. Methodology

Chemicals: WGO was procured from Wadi Al-Nahil, Riyadh, Saudi Arabia; TAA was obtained from Sigma Aldrich (St. Louis, MO, USA); olmutinib was from Weihua Pharma Co., Limited (Hangzhou, Zhejiang, China); and the ELISA kits for the inflammatory studies were procured from Thermo Fisher Scientific (Waltham, MA, USA).

Animal Protocol: Thirty male mice 4–6 weeks of age were randomly grouped into two groups: six in Group I, the control group (saline), and twenty-four mice in the other groups, which were given thioacetamide (0.3% in drinking water) for two weeks to induce liver and kidney damage. The 24 animals in the latter group were further divided into four groups (Groups II–V). Group II continued on thioacetamide (0.3% in water) for an additional five days; Group III received olmutinib at 30 mg/kg of their body weight (b.o.), per oral (p.o), for five days; Group IV received WGO (1400 mg/Kg) for five days (p.o); and Group V received olmutinib (50 mg/kg b.w, p.o) with WGO (1400 mg/Kg) for five days. The animals were kept at the animal care unit at the college of medicine, King Saud University, and were sacrificed by carbon dioxide asphyxiation following the guidelines of NC3R. The institutional animal ethics committee approved this research under ethical reference number KSU-SE-21-08.

### 2.1. Histopathology

Slices of the left liver lobe and left kidney from three animals in each group were fixed in 10% formalin for 24 h and later embedded as 5–6 mm sections in paraffin. Briefly, the samples were processed as follows: fixation in a 10% neutral formalin, block preparation in paraffin, cutting off 5–6 μm thick sections, and staining with hematoxylin–eosin (Leica Biosystems, Deer Park, IL, USA). These sections were later photographed and analyzed by an expert pathologist using an electron microscope (Leica Biosystems, USA). The pathologist had no information about the samples or treatment of experimental groups. All alterations from the standard structure were analyzed to assess the histopathological changes.

### 2.2. Inflammatory Marker Estimations

The total protein concentrations in all groups (*n* = 6) were measured from a standard curve using bovine serum albumin and represented as mg per gram of tissue. According to the manufacturer’s instructions, the expression levels of IL-1β and IL-6 were determined by ELISA at an optical density of 450 nm and expressed as pg/mg of protein, as previously described [26].

### 2.3. Enzyme Marker Estimation

The levels of enzymatic markers ALT, AST, LDH, and CK were estimated using UV-kinetic diagnostic kits (United Diagnostics Industry (UDI, Dammam, Saudi Arabia). First, kit reagents were reconstituted in 3 mL of double-distilled water. Then, 1 mL from this reconstituted reagent was incubated at 37 °C for 2 min and mixed with 10 µL of the serum. After 1 min of temperature equilibrium, the ALT, LDH, and CK absorbances were recorded every minute for 3 min, and the absorbance of AST was recorded after every minute for 2 min at 340 nm against distilled water to determine ΔA/min. Briefly, the ALT, CK, and AST activities were proportional to the oxidation of NADH or a decrease in absorbance rate, and ALT activity was proportional to the reduction in NAD or increase in absorbance in the case of LDH. The enzyme activities (U/L) were calculated using an extinction coefficient of NADH as 6.22 × 103 × M^−1^ cm^−1^.

### 2.4. Statistical Analysis

The data for interleukins and enzymatic markers were analyzed in GraphPad Prism (Version 6.01, Prism) and represented as means ± SD (*n* = 6). Between-group differences were determined with one-way ANOVA. A probability value (*p*-value) less than or equal to (0.05) was considered significant.

## 3. Results

### 3.1. Inflammatory Marker Expression in Liver and Kidney

**Figure 1:**
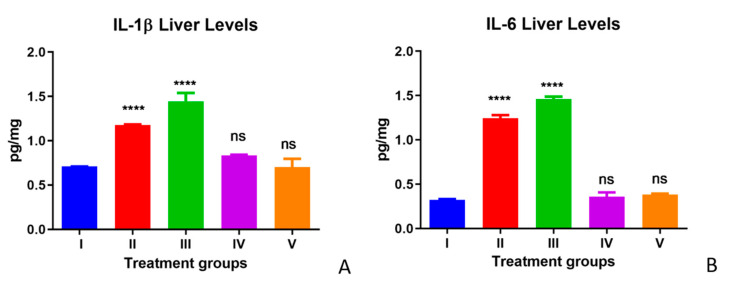
Effect of WGO and olmutinib on TAA-induced inflammation by IL-1β levels (**A**) and IL-6 levels (**B**) in the livers of mice groups; Group I (control liver), Group II (TAA with further TAA treatment), Group III (TAA with olmutinib treatment), Group IV (TAA treated with WGO), and Group V (TAA treated with olmutinib + WGO). Concentrations are expressed as pg/mg (*n* = 6); The treated groups were compared with the control group (**** *p* < 0.0001, ns: non-significant).

The IL-1β and IL-6 protein levels were measured to assess the inflammatory state with and without the treatments. Significant increases in IL-1β levels and IL-6 were observed in the TAA group compared with control group livers. The IL-1β and IL-6 levels observed in the olmutinib treatment group were slightly higher than in the TAA group. WGO supplementation reversed these marker levels in both Group IV (TAA-WGO) and Group V (TAA + olmutinib + WGO) compared with the control level. Post-treatment with WGO in mice did not show any significant changes in the levels of IL-1β and IL-6 compared with the control group, showing that WGO does not cause any type of inflammation or toxicity in liver tissue (Figure 1A,B).

**Figure 2:**
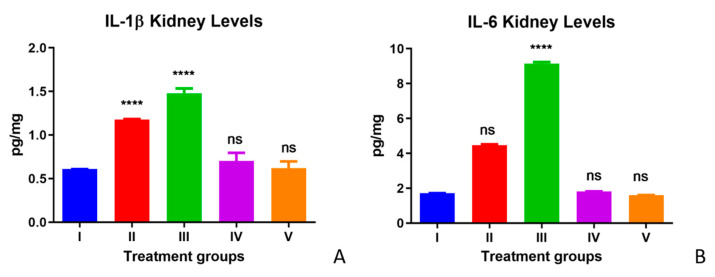
Effect of WGO and olmutinib on TAA-induced inflammation by IL-1β levels (**A**) and IL-6 levels (**B**) in kidneys of mice groups; Group I (control kidney), Group II (TAA with further TAA treatment), Group III (TAA with olmutinib treatment), Group IV (TAA treated with WGO), and Group V (TAA treated with olmutinib + WGO). Concentrations are expressed as pg/mg (*n* = 6). The treated groups were compared to the control group (**** *p* < 0.0001, ns: non-significant).

The IL-1β and IL-6 levels in kidney tissues are depicted in Figure 2A,B for all experimental Groups I–V. Significantly elevated levels of IL-1β levels and IL-6 were observed in the TAA-treated group of mice (Group II) in comparison with the control group (Group I). Olmutinib treatment to TAA-treated animals in Group III further increased the levels of IL-1β and IL-6 compared with the TAA-only group. Hence, the increased levels of IL-1β and IL-6 in Group III compared with those of Group II suggest that olmutinib potentiates TAA toxicity. The WGO alone group (Group IV) did not show any significant changes in the levels of IL-1β and IL-6, and their levels were similar to those of the control group (Group I). However, the elevated inflammatory marker levels were reversed by WGO supplementation in the olmutinib- and TAA-treated animals (Group V). The IL-1β and IL-6 levels in Group V were almost similar to the control group levels in Group I, suggesting a protective effect of WGO in mice against both TAA and olmutinib (Figure 2).

### 3.2. Enzyme Activity (AST, ALT, LDH, and CK)

**Figure 3:**
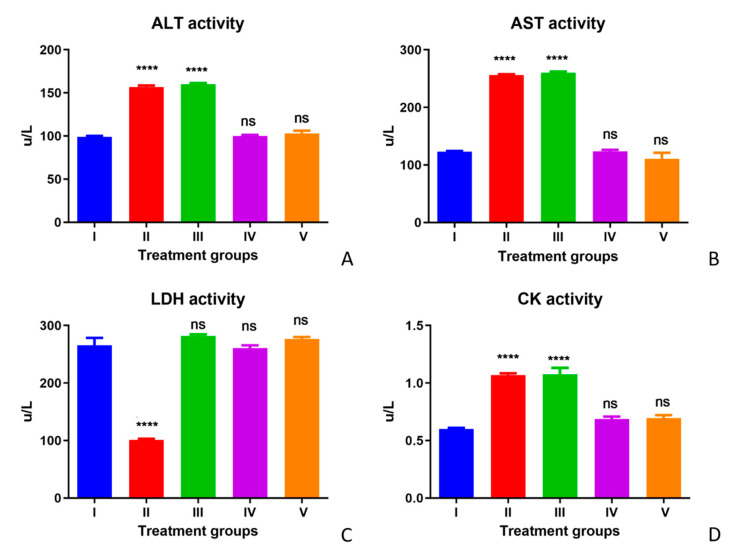
Effect of WGO and olmutinib on TAA-induced serum enzyme markers ALT (**A**), AST (**B**), LDH (**C**), and CK (**D**) in all mice groups; Group I (control), Group II (TAA with further TAA treatment), Group III (TAA with olmutinib treatment), Group IV (TAA treated with WGO), and Group V (TAA treated with olmutinib + WGO). Enzyme activities are represented as the amount of enzyme reducing one micromole of NAD for LDH and oxidizing one micromole NADH per min per liter (U/L) for AST, ALT, and CK. The treated groups were compared to the control group (**** *p* < 0.0001, ns: non-significant).

The enzyme activity (AST, ALT, and CK) in serum increased, whereas the LDH activity decreased significantly in Group II (TAA-treated group) compared with Group I (control group). The treatment with olmutinib (Group III) could not reverse the increased or decreased enzymatic activities, except for LDH in Group III, where olmutinib treatment reversed the LDH levels. In Group IV (the WGO group), no significant differences from the control group were observed. One of the hallmarks of oxidative stress is elevated energy consumption due to the high activity of antioxidant enzymes. Increased glucose uptake due to high energy demands leads to abnormal activity of LDH for converting glucose to lactate. The restoration of LDH in the olmutinib group could be due to resistance to drugs. The difference between Groups I, IV, and V was insignificant, showing the therapeutic effect of WGO with respect to enzymatic markers of serum in all these groups. A partial protective effect was observed in CK enzyme activities after treatments with WGO. Hence, WGO was assumed to be showing a partial protective activity with respect to CK serum enzyme activity levels (Figure 3A–D).

### 3.3. Histological Studies on Liver and Kidney

**Figure 4:**
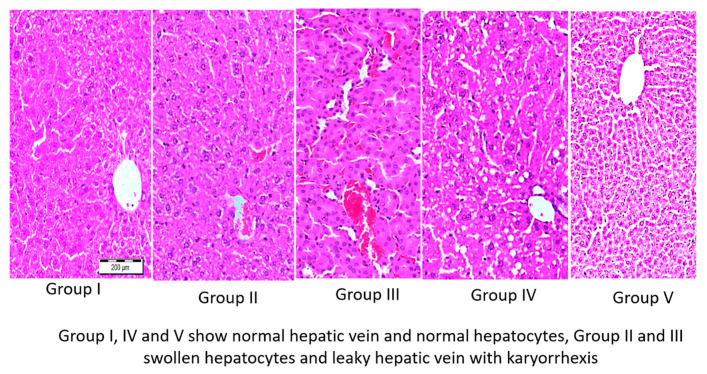
Images of H&E staining of livers of all experimental groups of mice (10×). Group I (control liver), Group II (TAA with further TAA treatment), Group III (TAA with olmutinib treatment), Group IV (TAA treated with WGO), and Group V (TAA treated with olmutinib + WGO). Groups I, IV, and V show normal hepatic vein and normal hepatocytes, while Groups II and III show swollen hepatocytes and leaky hepatic vein with karyorrhexis.

The TAA-treated Groups II and III showed similar degenerative histological changes after five days of their respective treatments. Group II was treated with TAA. Group III, treated with TAA followed by olmutinib, showed wrinkling of the hepatocyte cell membrane, leaky hepatic vein with karyorrhexis, and karyolysis of cells. The standard shape of the tissue was lost, and lobules’ disorganization was observed. Many lesions were seen due to cell and tissue degeneration. WGO alone (Group IV) and the co-treatment group (TAA + olmutinib + WGO) (Group V) were similar to the control group showing usual hepatic vein and normal hepatocytes with no lesions or karyolysis (Figure 4).

**Figure 5:**
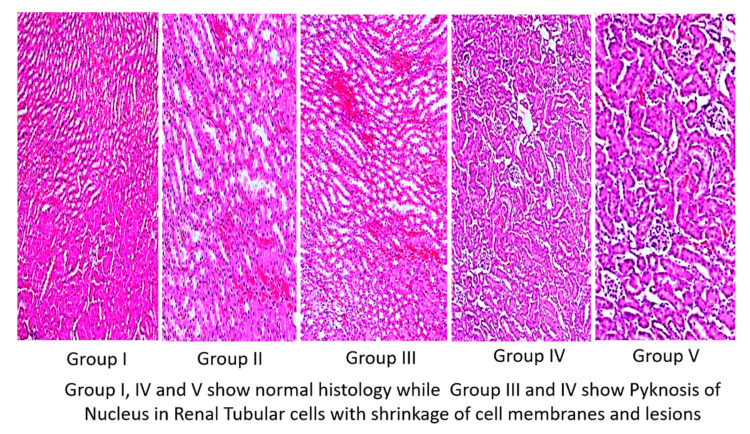
Images of H&E staining of kidneys of all experimental groups of mice (10×). Group I (control kidney), Group II (TAA with further TAA treatment), Group III (TAA with olmutinib treatment), Group IV (TAA treated with WGO), and Group V (TAA treated with olmutinib + WGO). Groups I, IV, and V show normal histology, while Groups II and III show pyknosis of the nucleus in renal tubular cells with cell membranes and lesion shrinkage.

Histopathological sections of the kidneys of all experimental groups are depicted in Figure 5. Groups I, IV, and V (Figure 5), the experimental mice groups, had normal cuboidal cells with a perfect nucleus and eosinophilic cytoplasm. The histology of kidneys treated with TAA (Group II) and TAA followed by olmutinib (Group III) showed an increase in hematopoiesis. In addition, there was a fusion of individual cell margins detected with fuzzy membranes of cells. Furthermore, vascular degeneration and pyknotic nuclei were observed in the TAA and olmutinib groups. WGO treatments reversed all of the degenerative changes in kidney tubules, with normal cuboidal cells having prominent nuclei.

## 4. Discussion

TAA is a potent hepatotoxic agent used widely to induce acute liver toxicity in an experimental rodent model by interfering in the nuclear transport of RNA from the nucleus to the cytoplasm and increases free radicals leading to membrane injury [27,28]. TAA is a potent hepatotoxicant and undergoes bioactivation to thioacetamide sulphoxide by microsomal enzyme CYP2E1 and thioacetamide-S, S-dioxide [28,29]. Cell signaling inhibitors, such as TKIs, are essential in chemotherapy treatments [30]. Olmutinib is approved to treat benign or metastatic lung cancers showing EGFR T790M mutation. This third-generation EGFR TKI treats non-small cell lung cancers [19,31]. Our results indicated that WGO significantly reversed TAA-induced effects in both kidney and livers of experimental groups, while olmutinib potentiated the toxicity induced by TAA. According to previous studies, WGO has antioxidant functions due to sterols, alpha-linolenic acid, vitamin E, and policosanols [32,33,34]. WGO-treated mice showed the restoration of elevated levels significantly in both liver and kidney relative to Group I (control group) and Group II (TAA assault group).

Moreover, a marked increase in inflammatory cytokine IL-1β and IL-6 levels were observed with TAA. Our study corroborates a previous study that stated that the activation of various signal pathways enhances downstream inflammation signals, leading to the further production of cytokines and chemotactic factors and, hence, the initiation of the oxidative reactions in the liver to fibrosis with TAA treatments [35,36]. Pro-inflammatory cytokines play a critical role in sustaining oxidative stress [37,38]. IL-1β is shown to be constitutively expressed to stimulate angiogenesis and to promote tumor growth and metastasis in some mouse melanoma models [39,40,41]. Similar to this study, progressive kidney damage and myeloma kidney development were reported in interleukin-6 transgenic mice [42]. The elevated levels of both inflammatory cytokines reversed in Group IV (TAA + WGO) and Group V (TAA + olmutinib + WGO) and were similar to the control group [43]. Higher levels of IL-6 were observed than IL-1β in the kidneys, and the reason for these elevated levels may be attributed to ADAM10 mediation, which slows constitutive IL-6R cleavage. The behavioral response of IL-6, either induced by a drug or due to inflammation, is highly complicated. In addition to pro-inflammatory behavior, it has an anti-inflammatory effect under certain disease conditions. Concurrent to our study, another study reported that IL-6 has a more active role in renal diseases than IL-1β [44]. This study reported the concomitant alteration of AST, ALT, LDH, and CK in serum based on TAA treatment, similar to previous literature. Group III, the olmutinib-treated group, showed a further increase in the IL-1b and IL-6 levels in both liver and kidney (more marked in IL-6 kidney level, as shown in Figure 2B) in the TAA-induced group (Group II). On the other hand, no difference was noticed in the ALT, AST, and CK activities between Groups II and III. The cytokine levels are produced in a cascade, which could be the reason for a marked increase in cytokine levels. An aggressive inflammatory response with the increased secretion of pro-inflammatory cytokines is attributed to the cytokine storm.

This study reports WGO as a protective agent against liver and kidney damage in mice. Previous studies showed the protective effects of WGO against streptozotocin, deltamethrin, and chlorpyriphos [45,46]. To the best of our knowledge, no study reported that the supplementation of WGO might be a promising strategy for managing or decreasing the side effects of chemotherapeutic drugs such as olmutinib and TAA. Wheat germ extract has been reported to have an influential role in enhancing the activity of the immune system, such as stimulating NK cell activity by reducing the expression of MHC I molecules; by stimulating TNF secretion of the macrophages; and by increasing ICAM 1 molecule expression on the vascular endothelial cells, which leads to the apoptosis of tumor cells as a consequence [47].

The inhibition of pro-inflammatory cytokines is the most critical function since these cytokines have essential roles in initiating the inflammatory response. The histological changes characterized the role of olmutinib in liver and kidney injuries. Furthermore, they also showed the protective role of WGO against this damage caused by TAA and olmutinib. Similar histological anomalies in liver-like wrinkling of the hepatocyte cell borders; leaky hepatic vein with karyorrhexis; karyolysis of cells; disorganization of lobules; and increased hematopoiesis with a fusion of cell membranes with fuzzy boundaries, vascular degeneration, and pyknotic nuclei in the kidney have been reported upon inductions with hexachlorocyclohexane [48,49].

## 5. Conclusions

The present study investigated the effect of WGO and olmutinib on the TAA toxicity mouse model using various biochemical, inflammatory markers, and histological studies. Our results indicated that olmutinib potentiated the toxic effects of TAA, whereas WGO had a protective effect against the toxicity induced by both TAA and olmutinib. We conclude that WGO is a non-toxic supplement that can reduce liver and kidney damage caused by TAA and olmutinib in mice.

## Data Availability

Data will be available on request to corresponding author.
